# Reshaping surgical specialist training in small animal surgery during and after the COVID‐19 pandemic

**DOI:** 10.1111/vsu.13660

**Published:** 2021-05-25

**Authors:** Akash Alexander, Heidi Radke

**Affiliations:** ^1^ Department of Veterinary Medicine University of Cambridge Cambridge UK

## Abstract

**Objective:**

To assess the perceived effects of the COVID‐19 pandemic on small animal surgical specialist training, among trainees and supervisors and to propose changes, based upon the results, that could be incorporated into training programs.

**Study Design:**

Anonymous online questionnaire survey.

**Sample Population:**

Eighty‐one eligible responses were collected in September 2020, including 52 European College of Veterinary Surgeons (ECVS) residents and 29 ECVS Diplomates acting as supervisors.

**Methods:**

Descriptive statistics were used to analyze the data. Fisher's exact test was used to test for significance.

**Results:**

A reduction in surgical case load was reported by 82% (*n* = 66/81) of respondents, with 82% (*n* = 54/66) of those believing that COVID‐19 had a mild‐to‐moderate impact on training. Compared to supervisors, residents were less likely to feel that appropriate guidance, a safe working environment, and measures to preserve training had been provided (*p* < .01). Only 45% (*n* = 22/49) of residents reported confidence with performing teleconsultations. Ninety percent (*n* = 73/81) of respondents considered online “case presentations” and “edited surgical video footage” as a positive ancillary tool.

**Conclusion:**

COVID‐19 has resulted in a reduction in case load and training for the majority of residents. A discrepancy between the opinions of residents and supervisors was noted on various aspects of COVID‐19 related effects.

**Impact:**

Open communication, as well as the use of additional training tools through digital platforms may help to preserve safe and effective training during times of decreased clinical activity. While this study has focused on surgical specialist training, the results could be applied to other disciplines.

## 1 INTRODUCTION

The coronavirus (COVID‐19) pandemic has had a widespread impact on healthcare services across human and veterinary medicine. A significant reduction of clinical case load, poorer training experience, and innovative practices have been reported from various sectors of post graduate medical education, including residency programs.^1^, ^2^, ^3^, ^4^, ^5^, ^6^ Veterinary medicine is considered a high‐risk profession, not only due to the typically high volume of client interactions but also due to the close proximity working environment. Despite this, the provision of veterinary care was considered “essential” during the pandemic and the vast majority of veterinary centers continued to work,^7^, ^8^ albeit with the implementation of extra‐ordinary operating procedures^9^ like use of enhanced personal protective equipment, implementation of social distancing, reduced staff numbers amongst others.

As seen in the human sector,^2^, ^4^, ^6^ it is likely that the above restrictions could result in an unpredictable gap in experience and an impact on specialist training, especially for residents on a fixed term training program such as a European College of Veterinary Surgeons (ECVS) approved residency program. With the uncertainty caused by additional waves and mutations of the virus,^10^, ^11^, ^12^ a complete return to normal operating procedures is not anticipated in the near future leading to significant restrictions in clinical and surgical opportunities for surgical residents. Furthermore, there will likely to be a continuing impact on the provision of conventional educational delivery tools, such as lectures and small group teaching sessions.

Whil there is some published literature reviewing the impact of COVID‐19 on human medical training, we recognized an absence of studies focused on veterinary surgical training. We felt that evaluating and understanding the impact of COVID‐19 in a timely fashion would be helpful to either mitigate or correct any adverse effect on training. In light of this, we designed an online questionnaire survey, of ECVS small animal surgery residents and resident supervisors. The survey aimed to assess the changes in clinical practice and perceptions of the impact of those changes on surgical training, as a result of COVID‐19. This information could then be used to inform potentially beneficial strategies to incorporate into specialist surgical training programs. Some innovative practices could also be sustained in the long‐term to help provide a progressive and modern approach to education.

## 2 MATERIALS AND METHODS

An anonymized 34 point survey was designed and produced on Google Forms (Appendix 1). The survey was piloted for content and usability by four ECVS residents and one ECVS resident supervisor. The survey was then distributed via the ECVS small animal email mailing list to all registered specialists and residents in September 2020. Responses were collected over a 4‐week period. Additional recruitment was performed via social media (LinkedIn, Facebook, and WhatsApp messenger). Participation in the survey was voluntary and no financial or other incentive was provided for completion.

An initial question regarding the position of the respondent (resident/supervisor/neither) was used to navigate to a set of personalized questions for each demographic. Respondents who answered “neither” had their survey terminated and were excluded from the study. Residents were asked questions about their own specific residency and the impact of COVID on themselves. The questions provided to the supervisors were identical in principle but worded to enquire about the impact on their resident's or residents' training program.

Questions were divided into three sections investigating: (1) background information and demographics, (2) changes of residency training during COVID‐19 pandemic, and (3) impact of changes due to COVID‐19. Respondents were asked to answer based on the time period between March and August 2020. Questions were closed‐ended, but where appropriate allowed respondents to provide a free text response or comment. Unless influenced by a previous response, all questions required a response. Where no response was provided for a question the denominator for the group size was adjusted, and stated, to allow for descriptive statistics. Upon completion of the survey, respondents were further offered the opportunity to provide any information or comments via an open‐ended question with free text entry.

Respondents consented to the storage and usage of the data contained within their responses for the purposes of this study. Ethical approval was granted by the Ethics and Welfare Committee at the Department of Veterinary Medicine, University of Cambridge (No: CR449).

Data were collated into Excel (V.16.36; Microsoft, Redmond, Washington) and analyzed in Prism 8 for Windows (V.8.4.2; GraphPad Software, San Diego, California). Data were assessed graphically for normality. Median and range was reported for skewed data. Categorical data were reported as percentages. Fisher's exact test was used to test the statistical significance of observed differences; *p* < .05 was considered significant.

## 3 RESULTS

Content and usability of the final survey were deemed appropriate by all five respondents involved in the pilot study. Responses obtained during the pilot study were not included in the final study data set; however, participants were invited again as part of the anonymized study.

### 3.1 Background and demographics

Eighty‐six responses for the survey were received, comprising 52 residents (30.8% response rate, based upon 169 residents currently in training), 29 resident supervisors and 5 who were “neither,” the latter of which were subsequently excluded from the study. The total number of resident supervisors enrolled with the ECVS was unavailable. The lowest possible response rate was deemed to be 17.2% (29/169) based even on a 1:1 ratio of residents to supervisors. Forty‐seven (58.0%) respondents were from private centers and 34 (42.0%) were from academic institutes. The median number of years since obtaining a degree in veterinary medicine was seven (0–23) and 19 (9–31) years for residents and supervisors, respectively. Standard veterinary surgery residency program (SVSRP), which consist of a minimum of 3 years training at a single institution fully supervised by an ECVS diplomate, made up 90% (*n* = 73/81) of responses. Dual‐site/co‐supervised SVSRP, which comprise of training split between two institutions made up 8.64% (*n* = 7/81) of responses. One respondent was undertaking an Alternate Veterinary Surgery Training Program (AVSTP). An AVSTP consists of spending the equivalent of at least 60% of three full time years working under direct supervision of an ECVS Diplomate. Sex distribution was equal in both groups (residents: 26 male and 26 female; supervisors:14 male and 15 female).

### 3.2 Changes of residency training during COVID‐19 pandemic

**FIGURE 1:**
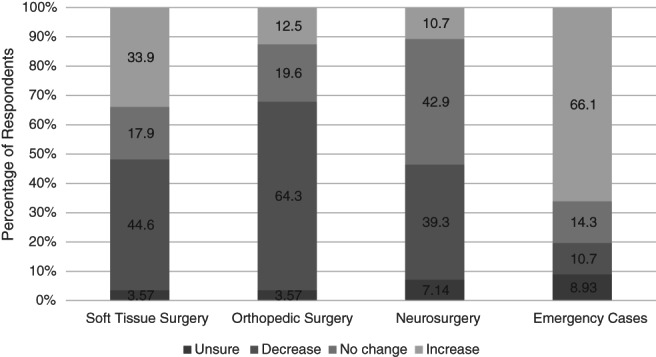
Perceived change in composition of case load seen by surgical residents between March 2020 and August 2020, compared to prior to March 2020

A change in the working schedule of residents was reported by 76.5% (*n* = 62/81) of all respondents, with government issued mandates affecting overall clinical practice of 75.3% (*n* = 61/81) of respondents. Eighty‐two percent (*n* = 66/81) of respondents reported a decrease in surgical load, with a quarter estimating a reduction by at least 50%. In contrast, 7.41% (*n* = 6/81) of respondents experienced an increased surgical load (of 20%–30%) between the months of March 2020 and August 2020. No change was reported by 11.1% (*n* = 9/81) of respondents. A change in the composition of cases seen, compared to that expected, was reported by 69.1% (*n* = 56/81). An increase in emergency cases was reported by 66.1% (*n* = 37/56) of those that reported a change. More respondents reported a decrease in orthopedic cases (64.3%, *n* = 36/56 compared to the reported decrease in soft tissue) and neurosurgery cases (44.6% and 39.3% respectively). An increase in soft‐tissue case load was also reported in 33.9% (*n* = 19/56; Figure 1). Telemedicine and virtual clinic were being performed by 35.8% (*n* = 29/81) of residents. Fifty‐five percent (*n* = 27/49, three nonresponders) of residents stated that they were “not confident” with conducting telemedicine consultations. Thirty‐one percent (*n* = 9/29) of supervisors stated that their residents felt confident with performing telemedicine. Residents already performing teleconsultations in response to the pandemic felt more confident, compared to residents who were not currently performing teleconsultations (*p* = .0005).

With regards to the structured education aspects of residency programs, residents reported a gap in didactic teaching more frequently than resident supervisors (53% [*n* = 28/52] vs. 27.6% [*n* = 8/29], *p* = .04)). Attendance of regular internal web‐based teaching (journal club, book club, etc.) was reported by 66.7% (*N* = 54/81) of respondents. Change of attendances at these sessions compared to pre‐COVID times were comparable with equal numbers reporting an increase (22%—18/81) or decrease (22%—18/81) in attendance. An increased attendance of externally provided web‐based teaching by residents (continuing professional development/continuing education, etc.) was reported by 70.4% (*n* = 57/81) respondents. Simulation‐based training tools were only available to 11.1% (*n* = 9/81) of respondents.

### 3.3 Perceptions of impact of COVID‐19 on clinical practice

**FIGURE 2:**
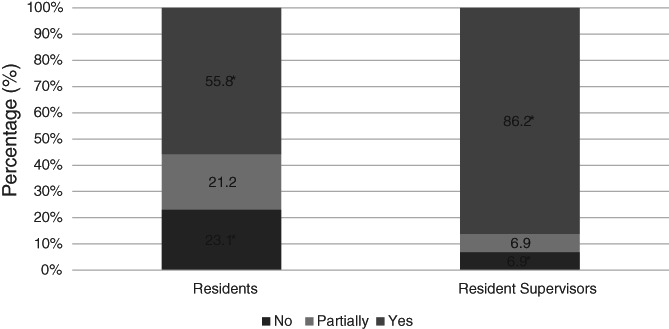
Percentage of residents (*n* = 52) and supervisors (*n* = 29) who think their institution has provided appropriate guidance on working during the pandemic. * Denotes statistical significance (*p* = .02)

**FIGURE 3:**
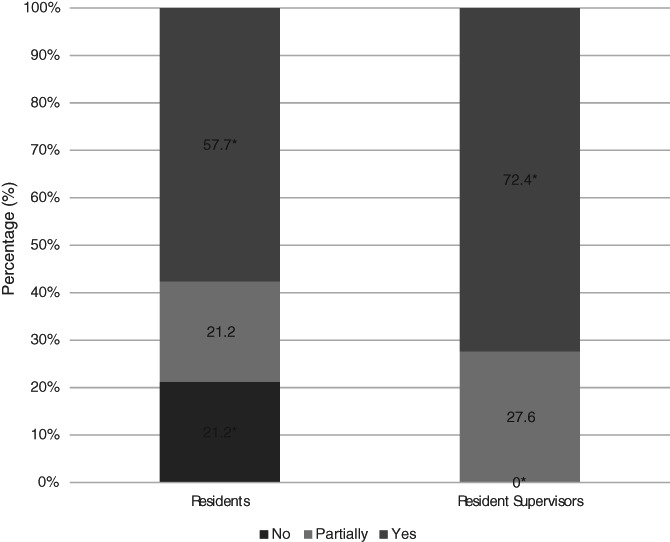
Percentage of residents who feel safe and well protected from COVID‐19 and supervisors who feel that their residents are safe and well protected. * Denotes statistical significance (*p* = .01)

Residents were less likely to feel that their institution had provided appropriate guidance on working during the pandemic compared with resident supervisors (55.8% vs. 86.2%, *p* = .02) (Figure 2). Residents were less likely to feel that a safe working environment had been provided, compared to resident supervisors (57.7% vs. 72.4%, *p* = .01) (Figure 3). Residents who were more than 5 years qualified or at private institutions were less likely to feel safe (52.6% vs. 71.4% and 55.9% vs. 61.1%, respectively). Forty‐two percent of female residents (*n* = 11/26) and 73.1% of male residents (*n* = 19/26) felt safe and protected from COVID‐19 at work (*p* = .04). There was no difference in the mean age or type of institution worked at between male and female residents.

### 3.4 Perceptions of impact of COVID‐19 on training program

Residents were less likely to feel that appropriate measures had been taken to preserve the effectiveness of their training by their institution, compared to resident supervisors (56.9% vs. 82.8%, *p* = .006) (Table 1). Fifty‐five percent (*n* = 18/33, one nonresponder) of residents at private institutions, compared to 61.1% (*n* = 11/18) at academic institutes, had felt that steps had been taken to preserve their training. Sixty‐eight percent (*n* = 17/25, one nonresponder) of residents at centers with three or more residents felt steps had been taken to preserve their training compared to 46.2% (*n* = 12/26) at centers with two or less residents.

**TABLE 1:** Percentage of respondents who believe that their institution has taken measures to preserve the effectiveness of resident training

	Yes (%)	Partially (%)	No (%)
Residents (*N* = 51, 1—prefer not to answer)	56.9*	15.7	27.5*
Resident supervisors (*N* = 29)	82.8*	13.8	3.45*

^*^ Denotes statistical significance (*p* = .006).

The use of web‐based teaching sessions, including case presentations, was seen as potentially helpful in the clinical training of surgical residents by 90.1% (*n* = 73/81) of respondents, with 95.1% (*n* = 77/81) of respondents feeling that live discussion around edited surgical videos could play a role in surgical training. Nearly half of respondents did not feel that remote supervision or the performing of virtual clinics and telemedicine would be effective in training and the gaining of clinical skills (Table 2).

**TABLE 2:** Percentage responses on the effectiveness of teaching methods on the training of surgical residents (*n* = 81)

	Yes (%)	No (%)	Not sure (%)
Can remote supervision be an effective part of residency training?	23.5	49.4	27.1
Do virtual clinics and telemedicine help to gain and/or improve clinical skills of residents?	34.5	42.0	23.5
Are web‐based teaching sessions, including case presentations, helpful in clinical training?	90.1	6.17	3.70
	Yes (%)	Partially (%)	No (%)
Could web‐based teaching replace traditional didactic teaching?	37.0	42.0	21.0
Could live discussion of edited surgical videos be helping in surgical training?	55.6	39.5	4.94
Would simulator training help residents to gain/improve surgical skills?	46.9	48.1	4.94

The impact of COVID‐19 on training programs to date was felt to be “mild” or “moderate” in 76.5% (*n* = 62/81) of cases while 12.3% (*n* = 10/81) of respondents felt to be “severe” or “catastrophic.” Only 3.70% (*n* = 3/81) predicted a “severe” or “catastrophic” impact on their future training (Table 3). Sixty percent (*n* = 9/15), of respondents who had experienced a decrease in clinical activity of 50% or more, deemed the long‐term impact of COVID‐19 to be moderate or severe compared to 21.2% (*n* = 14/66) in those who did not. It was more likely for respondents to predict an adverse impact if they had a significant reduction of workload compared to those who did not have a reduction in workload (66% vs. 21.2%). Respondents who were using internal web‐based teaching deemed the long‐term impact of COVID‐19 to be negligible or mild, with a higher frequency, compared to those not utilizing these tools (88.4% vs/ 70.4%). Seventy‐seven percent (*n* = 63) of respondents (38/52 residents and 25/29 supervisors) did not feel that COVID‐19 would result in an irreversible gap in resident training. Although half of the respondents (27/52 residents and 12/29 supervisors) described an extension of training programs as “pointless,” 37% (*n* = 30/81) of respondents (18/52 residents and 12/29 supervisors) considered an extension of training programs as “reasonable” or “necessary.”

**TABLE 3:** Perceived impact of COVID‐19 on surgical residency training to date and in the future during a long‐term period of co‐existence with the virus

	Perceived impact of COVID on residency training to date (*n* = 81) (%)	Perceived impact of COVID on residency training due to phase of co‐existence with virus (*n* = 80) (%)
No impact	11.1	22.5
Mild	45.7	48.8
Moderate	30.9	25.0
Severe	11.1	3.75
Catastrophic	1.23	0

Of the free text responses provided at the end of the survey, notable comments included the impact that COVID‐19 has had on the ability to complete additional training elements (*n* = 3) (e.g., rotations in other disciplines) and presentations at conferences (*n* = 2). One respondent expressed concern over a lack of direction and communication from the overarching training organization.

## 4 DISCUSSION

As the global pandemic took hold in early 2020, many institutions were faced with having to make adjustments and strategies for dealing with a crisis, on a scale never previously experienced. This study represents the first insight into the immediate effects that the COVID‐19 pandemic has had on the training of ECVS small animal surgery residents. Utilizing both the viewpoints of residents and resident supervisors, we aimed to highlight some of the challenges faced, the measures implemented and their perceived effectiveness. Compiling this information, we aimed to highlight key points that can be used to re‐shape and develop strategies to ensure the quality of small animal specialist training in the wake of this global crisis.

A specialist training program presents a distinctive challenge when faced with a period of restricted activity or change in working schedule due to its fixed term nature. The majority of residencies performed in small animal surgery are either 3 or 4 years long and as shown by the results of our study, over 90% of respondents saw a decrease in their surgical case load during the months of March 2020 and August 2020. This period, which is only the period of time elapsed during the first wave of the pandemic, could represent as much as one‐sixth of a residency program. The change in composition of cases seen may also have a significant effect on the experienced gained by residents. Awareness of the decrease in routine work, particularly orthopedics, should highlight topics for residents and supervisors to focus on with structured teaching. The increased emergency case load may lead to multiple pressures placed upon a residency, potentially impacting training. This may either be due to the emergent nature of the conditions and the need for efficient management of time pressures or due to the conflict of case timings with structured teaching. The impact of emergency work, particularly during unsocial hours, may have an impact on residents' ability to work effectively. There is potential for this to lead to increased work‐related stress and adversely impact mental health. Although we did not address mental health in this study, it is well reported in the human literature. Awareness of this as a risk factor is important for early identification of concerns among team members.

A striking pattern that emerged in the responses we received was a discrepancy between supervisors' and residents' perceptions of changes implemented to preserve training, their impact on resident safety and delivery of guidance. The perception of supervisors has yet to be investigated in human medicine with regards to the impact of COVID‐19 on training programs. However, as seen ubiquitously throughout medicine, good communication and balanced perceptions are fundamental to any working environment, particularly when presented with challenging situations. The differences between the opinions of supervisors and residents are likely multifactorial in nature and will often involve specific nuances unique to an individual center. It is likely that supervisors will have been involved in decision making at a managerial level that will have explored means of providing a safe environment for resident teaching. But if residents are neither aware of, nor involved in these discussions, and are simply faced with the final decisions, they may be more likely to feel that all options were explored, to preserve their training. Naturally, there will always be degrees of compromise involved in these steps and as adult learners, residents will accept this, as long as there is good communication about shared goals. We hope that the findings in this study highlight an awareness, to both residents and supervisors to engage together, particularly where concerns are present, to ensure that an optimal working environment is achieved. This can often be difficult, or a daunting prospect; however, this unified approach may be fundamental to ensuring effective training is maintained in an environment that feels safe for all parties involved. Accounting for social and personal factors including mental health, social stigma, and personal health requirements can help to provide a tailored, cohesive, and safe environment.^13^, ^14^, ^15^ As demonstrated in our results, residents who felt that measures had been taken to provide a safe working environment, were more likely to also respond that steps had been taken to preserve the effectiveness of their training. The use of government mandated guidelines regarding the use of personal protective equipment, coupled with considerations of unique individual circumstances are required to provide an environment that will be deemed safe. It is possible that these implementations will also have to change in response to the wider COVID‐19 situation, such as additional waves and concerns regarding new variants.

Social distancing and reducing face‐to‐face contact, a key intervention in reducing the transmission of COVID‐19,^16^ have led to nearly half of residents now performing telemedicine consultations. One sector in which great change has been witnessed in response to the pandemic, is the unified communications (UC) platform, which has developed with incredible speed.^17^ Platforms such as Zoom, Skype, and Microsoft Teams have been utilized for remote video consultation and internal meetings. However, our study revealed a general lack of confidence by residents in performing teleconsultations, which was largely expected as this has not been extensively utilized by veterinary professionals to date.^18^, ^19^ Despite historic concerns regarding the use of telemedicine in veterinary medicine,^20^ there is a strong possibility that even after COVID‐19 has passed, telemedicine may continue to be employed in veterinary medicine. While the number of residents currently undertaking telemedicine may seem lower than expected, there is potential for this number to increase as long‐term measures are implemented. Therefore, the acquisition of appropriate communication skills, particularly those regarding difficult conversations that may seem daunting over a digital platform, must be considered as an important feature in resident training. Training with simulated patients has been positively received by medical students and can often be provided remotely, allowing these skills to be developed even during times of enhanced social distancing.^21^, ^22^, ^23^ The development of these skills will also be transferrable to other aspects of resident training, including presentations conducted at virtual congresses. Abstract presentations, including at an international level, make up an element of the ECVS resident curriculum. With the restrictions placed on travel, virtual congresses have become commonplace and is likely to continue. Confidence in being able to present and communicate across UC platforms must not be underestimated, as it will contribute to multiple elements of a resident's training.

A large proportion of a small animal surgical residency is the practical aspect of gaining surgical experience under supervision. While alternatives do exist for providing ex‐vivo training, the majority of respondents felt that this would not, or would only partially help gain clinical skills and experience. While simulator‐based training has been shown to improve clinical skills, particularly in fields such as laparoscopy,^24^, ^25^, ^26^ they are seldom available to residents at their institutions. The above strategies could contribute toward the maintenance of skills during periods where a dramatic drop in case load is experienced, and provision should be considered by institutions. Furthermore, the use of these tools could continue to prove beneficial in the long term, even after the pandemic. There was great positivity toward the use of web‐based teaching sessions, such as case presentations, with a majority of respondents also stating that edited surgical videos paired with live discussion would be beneficial to training. The acquisition and provision of material are key to constructing these learning tools and should be seen as a task to implement as soon as possible. Although not novel, the author's institution has recently started to use sterile cases for camera phones to allow for intra‐operative photography and videography to be performed. This allows for the capture of higher quality footage, compared to a nonsterile assistant attempting to capture images from a distance. This could be taken even further with the use of head mounted videography to give trainee surgeons a unique “surgeon's‐eye” perspective. The success of this tool has recently been shown in human medicine^6^, ^27^, ^28^, ^29^, ^30^ and has shown early promise in the veterinary field.^31^ The use of an integrated audio‐visual platform in surgical theaters could prove to be a versatile tool in the long‐term adaptations to COVID‐19. Smart glasses, such as Google Glass, have already been trialed in human medicine and were well received in certain applications. They have displayed improved surgical workflow and provided an opportunity for remote supervision and enhanced surgical education.^32^ Their application has also been seen within the pandemic efforts in facilitating virtual ward rounds, which were deemed feasible, effective, and widely accepted as an alternative, under the circumstances.^33^

Application of remote or web‐based learning must be targeted to ensure it meets the expectation and requirements of residents. As demonstrated in our results, these methods will not be able to replace the practical and in vivo experience of surgery. However, when utilized in settings such as the training of nontechnical skills, including, but not limited to clinical decision making, surgical planning, procedural guidance and post‐operative care, they could be a great complement to the curriculum.^6^ Interestingly, our results highlighted that despite a general decrease in clinical activity, there was also a decrease in residents' attendance to structured educational aspects of the residency by nearly a quarter. Supervisors should try to identify and rectify decreased attendance by an individual or inadvertent cessation of teaching sessions to ensure that training opportunities continue. Reasons for decreased attendance are likely unique to individuals, but the influence of personal factors such as social isolation, anxiety and mental health should be considered. Awareness of these factors can help supervisors and co‐residents assist their colleagues during these times and highlight concerns that may otherwise go unnoticed.

Whil positive influences can be evident, it is important to recognize potential concerns that may arise as a result of implemented changes. Our survey highlighted a concern with the provision of remote supervision to residents, with the majority of respondents being unsure about or against it in principle, a finding echoed in the study by Ferrara et al..^6^ We believe that the provision of in‐person mentoring delivered in a socially distanced manner for residents is imperative. Another concern was the impact that COVID‐19 restrictions has had on the ability of residents to attend externships to complete rotations in other disciplines. Government mandated travel and hospitality restrictions may contribute to residents' inability to travel and remain in a location while completing externships. As the co‐existence phase with the virus continues, consideration of how centers can safely and effectively allow visitors and allow their residents to visit other hospitals, must be considered early to prevent ongoing disruption to training. The use of rapid, point of care testing will likely be highly beneficial in achieving this. The advent of low‐cost testing in the future may help to mitigate the initial expense associated with increased testing. This, coupled with the ability to protect hospitals from outbreaks of COVID‐19 that may itself result in dramatic reductions in capacity, may prove testing to be cost efficient overall.

As seen throughout the pandemic, individuals have turned to governing bodies for guidance on dealing with and adapting to the impact of COVID‐19. To be able to effectively advise and recommend changes, data and research must be available upon which this guidance can be based. To date, veterinary literature has lacked this research and as a result it may have been difficult for sound recommendations to have been made and communicated. We hope that the results in this study will contribute toward and inform governing bodies to evaluate the impact of the pandemic and in future planning of strategies. We believe that the early implementation of proposed strategies discussed in this article is crucial to ensure continued provision of high‐quality training, particularly in light of a long‐term co‐existence with the virus.

Limitations of the study were consistent with those often seen in survey‐based questionnaires. The response rate has yielded a relatively small sample size but is in keeping with expectations of online surveys. The survey was also not able to discriminate or pair responses that were arising from one center with multiple residents, or residents whose supervisors also responded, to allow for direct comparison of perceptions implemented at one institution. This may have also resulted in overlap of responses, causing a falsely high response rate to certain questions. Responses presented in the survey could also be affected by a negative bias with respondents who have concerns or problems with their residency being more likely to complete the survey. Therefore, the concerns raised may be unique to an individual and less applicable to a larger audience. This is particularly important to consider with regards to the free text responses. Differences in the management of the COVID‐19 pandemic by individual governments mean that regional and international variations likely exist and must be considered by the reader when applying the findings to their particularly setting. Due to the anonymous nature of the survey and availability through social media platforms, it is conceivable that unscrupulous individuals may have completed the survey more than once or may have not been a resident or supervisor. Finally, this study did not feature a negative control to assess the perception of residency training prior to COVID‐19. It is plausible that insufficiencies in training may have been present, but only now have been brought to light. Additional studies are imminently needed to continue assessing the ongoing impact of this global crisis on the veterinary profession in general and training in particular. Longitudinal studies assessing the long‐term effect of educational disruption would also ensure that the training of tomorrow's specialists is not hampered and the excellent provision of care to animals can be continued in the wake of COVID‐19.

Whil this study has focused on the specialist training of ECVS small animal surgical residents, we hope that the findings of this study can be made applicable to readers in different educational settings, from those in other specialist disciplines, those undertaking certificate level studies and even undergraduate veterinary and veterinary nursing training.

In conclusion, the COVID‐19 pandemic has provided a unique challenge to small animal surgical residency training. Open communication between supervisors and residents remains key to ensuring a safe and effective working environment. Additionally, the implementation of ancillary training tools through modern digital platforms may help to preserve skills and allow clinical experience to be gained during times of decreased clinical activity. Some of the suggestions presented here, including the use of videography and case‐based presentations, may provide a great medium for learning even after the pandemic.

## CONFLICT OF INTEREST

The authors declare no conflict of interest related to this report.

## AUTHORS CONTRIBUTION

Akash Alexander and Heidi Radke were involved in the conception and design of the study, including acquisition, analysis, and interpretation of the data. They drafted and revised the manuscript and gave approval of the version to be published.

## Supporting information


**Appendix S1**: Supporting Information
