# Supplementary material for: Reshaping surgical specialist training in small animal surgery during and after the COVID‐19 pandemic
Source: Vet Surg. 2021 May 25;50(5):924–32. doi: 10.1111/vsu.13660 (PMC8242755; doi:10.1111/vsu.13660)
Supplement: Supplementary file 1 — Appendix S1: Supporting Information [file VSU-50-924-s001.pdf]

VETERINARY SURGERY

Reshaping Surgical Specialist Training in Small Animal Surgery During and After the COVID-19 Pandemic

Journal:	*Veterinary Surgery*
Manuscript ID	VSU-21-049.R1
Manuscript Type:	Original Article - Research
Keywords:	Education, Distance Learning, ECVS, Training, Surgery, Coronavirus, COVID-19

SCHOLARONE™
Manuscripts

Title

Reshaping Surgical Specialist Training in Small Animal Surgery During and After the COVID-19 Pandemic

Authors

REDACTED

Author Affiliations

REDACTED

Corresponding Author

REDACTED

The authors received no funding or financial support towards the research of this study.

For Peer Review

Abstract

Objective: To assess the perceived effects of the COVID-19 pandemic on small animal surgical specialist training, amongst trainees and supervisors; and to propose changes, based upon the results, that could be incorporated into training programs.

Study Design: Anonymous online questionnaire survey.

Sample Population: Eighty-one eligible responses were collected in September 2020, including 52 European College of Veterinary Surgeons (ECVS) residents and 29 ECVS Diplomates acting as supervisors.

Methods: Descriptive statistics were used to analyze the data. Fisher's exact test was used to test for significance.

Results: A reduction in surgical case load was reported by 82% (n=66/81) of respondents, with 82% (n=54/66) of those believing that COVID-19 had a mild to moderate impact on training. Compared to supervisors, residents were less likely to feel that appropriate guidance, a safe working environment and measures to preserve training had been provided. ($P<.01$). Only 45% (n=22/49) of residents reported confidence with performing teleconsultations. Ninety percent (n=73/81) of respondents considered online 'case presentations' and 'edited surgical video footage' as a positive ancillary tool.

Conclusion: COVID-19 has resulted in a reduction in case load and training for the majority of residents. A discrepancy between the opinions of residents and supervisors was noted on various aspects of COVID-19 related effects.

Impact: Open communication, as well as the use of additional training tools through digital platforms may help to preserve safe and effective training during times of decreased clinical activity. Whilst this study has focused on surgical specialist training, the results could be applied to other disciplines.

For Peer Review

Introduction

The coronavirus (COVID-19) pandemic has had a widespread impact on healthcare services across human and veterinary medicine. A significant reduction of clinical case load, poorer training experience and innovative practices have been reported from various sectors of post graduate medical education, including residency programs [1–6]. Veterinary medicine is considered a high-risk profession, not only due to the typically high volume of client interactions, but also due to the close proximity working environment. Despite this, the provision of veterinary care was considered 'essential' during the pandemic and the vast majority of veterinary centers continued to work [7,8], albeit with the implementation of extra-ordinary operating procedures[9] like use of enhanced personal protective equipment, implementation of social distancing, reduced staff numbers amongst others.

As seen in the human sector [2,4,6], it is likely that the above restrictions could result in an unpredictable gap in experience and an impact on specialist training, especially for residents on a fixed term training program such as a European College of Veterinary Surgeons (ECVS) approved residency program. With the uncertainty caused by additional waves and mutations of the virus[10–12], a complete return to normal operating procedures is not anticipated in the near future leading to significant restrictions in clinical and surgical opportunities for surgical residents. Furthermore, there will likely to be a continuing impact on the provision of conventional educational delivery tools, such as lectures and small group teaching sessions.

Whilst there is some published literature reviewing the impact of COVID-19 on human medical training, we recognized an absence of studies focused on veterinary surgical training. We felt that evaluating and understanding the impact of COVID -19 in a timely fashion would be helpful to either mitigate or correct any adverse effect on training. In light of this, we designed

For Peer Review

an online questionnaire survey, of ECVS small animal surgery residents and resident supervisors. The survey aimed to assess the changes in clinical practice and perceptions of the impact of those changes on surgical training, as a result of COVID-19. This information could then be used to inform potentially beneficial strategies to incorporate into specialist surgical training programs. Some innovative practices could also be sustained in the long-term to help provide a progressive and modern approach to education.

For Peer Review

Materials and Methods

An anonymized 34 point survey was designed and produced on Google Forms (Appendix 1). The survey was piloted for content and useability by four ECVS residents and one ECVS resident supervisor. The survey was then distributed via the ECVS small animal email mailing list to all registered specialists and residents in September 2020. Responses were collected over a 4-week period. Additional recruitment was performed via social media (LinkedIn, Facebook and WhatsApp messenger). Participation in the survey was voluntary and no financial or other incentive was provided for completion.

An initial question regarding the position of the respondent (resident/supervisor/neither) was used to navigate to a set of personalized questions for each demographic. Respondents that answered 'neither' had their survey terminated and were excluded from the study. Residents were asked questions about their own specific residency and the impact of COVID on themselves. The questions provided to the supervisors were identical in principle but worded to enquire about the impact on their resident's or residents' training program.

Questions were divided into three sections investigating: (1) Background information and demographics, (2) Changes of residency training during COVID-19 pandemic, and (3) Impact of changes due to COVID-19. Respondents were asked to answer based on the time period between March – August 2020. Questions were closed-ended, but where appropriate allowed respondents to provide a free text response or comment. Unless influenced by a previous response, all questions required a response. Where no response was provided for a question the denominator for the group size was adjusted, and stated, to allow for descriptive statistics. Upon completion of the survey, respondents were further offered the opportunity to provide any information or comments via an open-ended question with free text entry.

For Peer Review

Respondents consented to the storage and usage of the data contained within their responses for the purposes of this study. Ethical approval was granted by the Ethics and Welfare Committee at the REDACTED.

Data were collated into Excel (V.16.36; Microsoft, Redmond, Washington) and analyzed in Prism 8 for Windows (V.8.4.2; GraphPad Software, San Diego, California). Data were assessed graphically for normality. Median and range was reported for skewed data. Categorical data were reported as percentages. Fisher's exact test was used to test the statistical significance of observed differences; $P < .05$ was considered significant.

For Peer Review

Results

Content and useability of the final survey was deemed appropriate by all 5 respondents involved in the pilot study. Responses obtained during the pilot study were not included in the final study data set, however participants were invited again as part of the anonymized study.

Background and Demographics:

Eighty-six responses for the survey were received, comprising 52 residents (30.8% response rate, based upon 169 residents currently in training), 29 resident supervisors and 5 that were 'neither', the latter of which were subsequently excluded from the study. The total number of resident supervisors enrolled with the ECVS was unavailable. The lowest possible response rate was deemed to be 17.2% (29/169) based even on a 1:1 ratio of residents to supervisors. Forty-seven (58.0%) respondents were from private centers and 34 (42.0%) were from academic institutes. The median number of years since obtaining a degree in veterinary medicine was seven (0-23) and 19 (9-31) years for residents and supervisors respectively. Standard veterinary surgery residency program (SVSRP), which consist of a minimum of three years training at a single institution fully supervised by an ECVS diplomate, made up 90% (n=73/81) of responses. Dual-site/Co-supervised SVSRP, which comprise of training split between two institutions made up 8.64% (n=7/81) of responses. One respondent was undertaking an Alternate Veterinary Surgery Training Program (AVSTP). An AVSTP consists of spending the equivalent of at least 60% of three full time years working under direct supervision of an ECVS Diplomate. Sex distribution was equal in both groups (residents: 26 male and 26 female; supervisors:14 male and 15 female).

Changes of Residency training during COVID-19 pandemic:

For Peer Review

A change in the working schedule of residents was reported by 76.5% (n=62/81) of all respondents, with government issued mandates affecting overall clinical practice of 75.3% (n=61/81) of respondents. Eighty-two percent (n=66/81) of respondents reported a decrease in surgical load, with a quarter estimating a reduction by at least 50%. In contrast, 7.41% (n=6/81) of respondents experienced an increased surgical load (of 20-30%) between the months of March 2020 and August 2020. No change was reported by 11.1% (n=9/81) of respondents. A change in the composition of cases seen, compared to that expected, was reported by 69.1% (n=56/81). An increase in emergency cases was reported by 66.1% (n=37/56) of those that reported a change. More respondents reported a decrease in orthopedic cases (64.3% (n=36/56 compared to the reported decrease in soft tissue and neurosurgery cases (44.6 % and 39.3 % respectively). An increase in soft-tissue case load was also reported in 33.9% (n=19/56 (Figure 1). Telemedicine and virtual clinic were being performed by 35.8% (n=29/81) of residents. Fifty-five percent (n=27/49, 3 non-responders) of residents stated they were 'not confident' with conducting telemedicine consultations. Thirty-one percent (n=9/29) of supervisors stated that their residents felt confident with performing telemedicine. Residents already performing teleconsultations in response to the pandemic felt more confident, compared to residents who were not currently performing teleconsultations (P=.0005).

For Peer Review

With regards to the structured education aspects of residency programs, residents reported a gap in didactic teaching more frequently than resident supervisors (53% [n=28/52] vs 27.6% [n=8/29], P=.04)). Attendance of regular internal web-based teaching (journal club, book club, etc.) was reported by 66.7% (N=54/81) of respondents. Change of attendances at these sessions compared to pre-COVID times were comparable with equal numbers reporting an increase (22%- 18/81) or decrease (22% - 18/81) in attendance. An increased attendance of externally provided web-based teaching by residents (continuing professional development/continuing

education, etc.) was reported by 70.4% (n=57/81) respondents. Simulation-based training tools were only available to 11.1% (n=9/81) of respondents.

Perceptions of impact of Covid-19 on clinical practice:

Residents were less likely to feel that their institution had provided appropriate guidance on working during the pandemic compared with resident supervisors (55.8% vs 86.2%, P=.02) (Figure 2). Residents were less likely to feel that a safe working environment had been provided, compared to resident supervisors (57.7% vs 72.4%, P=.01) (Figure 3). Residents who were more than five years qualified or at private institutions were less likely to feel safe (52.6% vs 71.4% and 55.9% vs 61.1% respectively). Forty-two percent of female residents (n=11/26) and 73.1% of male residents (n=19/26) felt safe and protected from COVID-19 at work (P=.04). There was no difference in the mean age or type of institution worked at between male and female residents.

Perceptions of impact of Covid-19 on training programme

Residents were less likely to feel that appropriate measures had been taken to preserve the effectiveness of their training by their institution, compared to resident supervisors (56.9% vs 82.8%, P=.006) (Table 1). Fifty-five percent (n=18/33, 1 non-responder) of residents at private institutions, compared to 61.1% (n=11/18) at academic institutes, had felt that steps had been taken to preserve their training. Sixty-eight percent (n=17/25, 1 non-responder) of residents at centers with three or more residents felt steps had been taken to preserve their training compared to 46.2% (n=12/26) at centers with two or less residents.

The use of web-based teaching sessions, including case presentations, was seen as potentially helpful in the clinical training of surgical residents by 90.1% (n=73/81) of respondents, with 95.1% (n=77/81) of respondents feeling that live discussion around edited surgical videos

For Peer Review

could play a role in surgical training. Nearly half of respondents did not feel that remote supervision or the performing of virtual clinics and telemedicine would be effective in training and the gaining of clinical skills (Table 2).

The impact of COVID-19 on training programs to date was felt to be ‘mild’ or ‘moderate’ in 76.5% (n=62/81) of cases whilst 12.3% (n=10/81) of respondents felt the to be ‘severe’ or ‘catastrophic’. Only 3.70% (n=3/81) predicted a ‘severe’ or ‘catastrophic’ impact on their future training (Table 3). Sixty percent (n=9/15), of respondents that had experienced a decrease in clinical activity of 50% or more, deemed the long-term impact of COVID-19 to be moderate or severe compared to 21.2% (n=14/66) in those that did not. It was more likely for respondents to predict an adverse impact if they had a significant reduction of workload compared to those who did not have a reduction in workload (66% vs 21.2%). Respondents who were using internal web-based teaching deemed the long-term impact of COVID-19 to be negligible or mild, with a higher frequency, compared to those not utilizing these tools (88.4% vs 70.4%). Seventy-seven percent (n=63) of respondents (38/52 residents and 25/29 supervisors) did not feel that COVID-19 would result in an irreversible gap in resident training. Although half of the respondents (27/52 residents and 12/29 supervisors) described an extension of training programs as ‘pointless’, 37% (n=30/81) of respondents (18/52 residents and 12/29 supervisors) considered an extension of training programs as ‘reasonable’ or ‘necessary’.

Of the free text responses provided at the end of the survey, notable comments included the impact that COVID-19 has had on the ability to complete additional training elements (n=3) (eg: rotations in other disciplines) and presentations at conferences (n=2). One respondent expressed concern over a lack of direction and communication from the overarching training organization.

For Peer Review

Discussion

As the global pandemic took hold in early 2020, many institutions were faced with having to make adjustments and strategies for dealing with a crisis, on a scale never previously experienced. This study represents the first insight into the immediate effects that the COVID-19 pandemic has had on the training of ECVS small animal surgery residents. Utilizing both the viewpoints of residents and resident supervisors, we aimed to highlight some of the challenges faced, the measures implemented and their perceived effectiveness. Compiling this information, we aimed to highlight key points that can be used to re-shape and develop strategies to ensure the quality of small animal specialist training in the wake of this global crisis.

A specialist training program presents a distinctive challenge when faced with a period of restricted activity or change in working schedule due to its fixed term nature. The majority of residencies performed in small animal surgery are either three or four years long and as shown by the results of our study, over 90% of respondents saw a decrease in their surgical case load during the months of March 2020 and August 2020. This period, which is only the period of time elapsed during the first wave of the pandemic, could represent as much as 1/6th of a residency program. The change in composition of cases seen may also have a significant effect on the experienced gained by residents. Awareness of the decrease in routine work, particularly orthopedics, should highlight topics for residents and supervisors to focus on with structured teaching. The increased emergency case load may lead to multiple pressures placed upon a residency, potentially impacting training. This may either be due to the emergent nature of the conditions and the need for efficient management of time pressures or the conflict of case timings with structured teaching. The impact of emergency work, particularly during unsocial hours, may have an impact on residents' ability to work effectively. There is potential for this

For Peer Review

to lead to increased work-related stress and adversely impact mental health. Although we did not address mental health in this study it is well reported in the human literature. Awareness of this as a risk factor is important for early identification of concerns amongst team members.

A striking pattern that emerged in the responses we received was a discrepancy between supervisors' and residents' perceptions of changes implemented to preserve training, their impact on resident safety and delivery of guidance. The perception of supervisors has yet to be investigated in human medicine with regards to the impact of COVID-19 on training programs. However, as seen ubiquitously throughout medicine, good communication and balanced perceptions are fundamental to any working environment, particularly when presented with challenging situations. The differences between the opinions of supervisors and residents are likely multifactorial in nature and will often involve specific nuances unique to an individual center. It is likely that supervisors will have been involved in decision making at a managerial level that will have explored means of providing a safe environment for resident teaching. But if residents are neither aware of, nor involved in these discussions, and are simply faced with the final decisions, they may be more likely to feel that all options were explored, to preserve their training. Naturally, there will always be degrees of compromise involved in these steps and as adult learners, residents will accept this, as long as there is good communication about shared goals. We hope that the findings in this study highlight an awareness, to both residents and supervisors to engage together, particularly where concerns are present, to ensure that an optimal working environment is achieved. This can often be difficult, or a daunting prospect, however this unified approach may be fundamental to ensuring effective training is maintained in an environment that feels safe for all parties involved. Accounting for social and personal factors including mental health, social stigma and personal health requirements can help to provide a tailored, cohesive and safe environment[14–16]. As demonstrated in our results, residents who felt that measures had been taken to provide a safe working environment, were more likely

For Peer Review

to also respond that steps had been taken to preserve the effectiveness of their training. The use of government mandated guidelines regarding the use of personal protective equipment, coupled with considerations of unique individual circumstances are required to provide an environment that will be deemed safe. It is possible that these implementations will also have to change in response to the wider COVID-19 situation, such as additional waves and concerns regarding new variants.

Social distancing and reducing face-to-face contact, a key intervention in reducing the transmission of COVID-19[17], has led to nearly half of residents now performing telemedicine consultations. One sector in which great change has been witnessed in response to the pandemic, is the unified communications (UC) platform, which has developed with incredible speed[18]. Platforms such as Zoom, Skype and Microsoft Teams have been utilized for remote video consultation and internal meetings. However, our study revealed a general lack of confidence by residents in performing teleconsultations, which was largely expected as this has not been extensively utilized by veterinary professionals to date[19,20]. Despite historic concerns regarding the use of telemedicine in veterinary medicine[21], there is a strong possibility that even after COVID-19 has passed, telemedicine may continue to be employed in veterinary medicine. While the number of residents currently undertaking telemedicine may seem lower than expected, there is potential for this number to increase as long-term measures are implemented. Therefore, the acquisition of appropriate communication skills, particularly those regarding difficult conversations that may seem daunting over a digital platform, must be considered as an important feature in resident training. Training with simulated patients has been positively received by medical students and can often be provided remotely, allowing these skills to be developed even during times of enhanced social distancing[22–24]. The development of these skills will also be transferrable to other aspects of resident training, including presentations conducted at virtual congresses. Abstract presentations, including at an

For Peer Review

international level, make up an element of the ECVS resident curriculum. With the restrictions placed on travel, virtual congresses have become commonplace and is likely to continue. Confidence in being able to present and communicate across UC platforms must not be underestimated, as it will contribute to multiple elements of a resident's training.

A large proportion of a small animal surgical residency is the practical aspect of gaining surgical experience under supervision. Whilst alternatives do exist for providing ex-vivo training, the majority of respondents felt that this would not, or would only partially help gain clinical skills and experience. While simulator based training has been shown to improve clinical skills, particularly in fields such as laparoscopy [26–28], they are seldom available to residents at their institutions. The above strategies could contribute towards the maintenance of skills during periods where a dramatic drop in case load is experienced, and provision should be considered by institutions. Furthermore, the use of these tools could continue to prove beneficial in the long term, even after the pandemic. There was great positivity towards the use of web-based teaching sessions, such as case presentations, with a majority of respondents also stating that edited surgical videos paired with live discussion would be beneficial to training. The acquisition and provision of material is key to constructing these learning tools and should be seen as a task to implement as soon as possible. Although not novel, the author's institution has recently started to use sterile cases for camera phones to allow for intra-operative photography and videography to be performed. This allows for the capture of higher quality footage, compared to a non-sterile assistant attempting to capture images from a distance. This could be taken even further with the use of head mounted videography to give trainee surgeons a unique "surgeon's-eye" perspective. The success of this tool has recently been shown in human medicine [6,29–32] and has shown early promise in the veterinary field[33]. The use of an integrated audio-visual platform in surgical theatres could prove to be a versatile tool in the long-term adaptations to COVID-19. Smart glasses, such as Google Glass, have already been

For Peer Review

trialed in human medicine and were well received in certain applications. They have displayed improved surgical workflow and provided an opportunity for remote supervision and enhanced surgical education[34]. Their application has also been seen within the pandemic efforts in facilitating virtual ward rounds, which were deemed feasible, effective, and widely accepted as an alternative, under the circumstances[35].

Application of remote or web-based learning must be targeted to ensure it meets the expectation and requirements of residents. As demonstrated in our results, these methods will not be able to replace the practical and in-vivo experience of surgery. However, when utilized in settings such as the training of non-technical skills, including, but not limited to clinical decision making, surgical planning, procedural guidance and post-operative care, they could be a great complement to the curriculum [6]. Interestingly, our results highlighted that despite a general decrease in clinical activity, there was also a decrease in residents' attendance to structured educational aspects of the residency by nearly a quarter. Supervisors should try to identify and rectify decreased attendance by an individual or inadvertent cessation of teaching sessions to ensure that training opportunities continue. Reasons for decreased attendance are likely unique to individuals, but the influence of personal factors such as social isolation, anxiety and mental health should be considered. Awareness of these factors can help supervisors and co-residents assist their colleagues during these times and highlight concerns that may otherwise go unnoticed.

Whilst positive influences can be evident, it is important to recognize potential concerns that may arise as a result of implemented changes. Our survey highlighted a concern with the provision of remote supervision to residents, with the majority of respondents being unsure about or against it in principle, a finding echoed in the study by Ferrara et al. [6]. We believe that the provision of in-person mentoring delivered in a socially distanced manner for residents

For Peer Review

is imperative. Another concern was the impact that COVID-19 restrictions has had on the ability of residents to attend externships to complete rotations in other disciplines. Government mandated travel and hospitality restrictions may contribute to residents' inability to travel and remain in a location while completing externships. As the co-existence phase with the virus continues, consideration of how centers can safely and effectively allow visitors and allow their residents to visit other hospitals, must be considered early to prevent ongoing disruption to training. The use of rapid, point of care testing will likely be highly beneficial in achieving this. The advent of low-cost testing in the future may help to mitigate the initial expense associated with increased testing. This, coupled with the ability to protect hospitals from outbreaks of COVID-19 that may itself result in dramatic reductions in capacity, may prove testing to be cost efficient overall.

As seen throughout the pandemic, individuals have turned to governing bodies for guidance on dealing with and adapting to the impact of COVID-19. To be able to effectively advise and recommend changes, data and research must be available upon which this guidance can be based. To date, veterinary literature has lacked this research and as a result it may have been difficult for sound recommendations to have been made and communicated. We hope that the results in this study will contribute towards and inform governing bodies to evaluate the impact of the pandemic and in future planning of strategies. We believe that the early implementation of proposed strategies discussed in this paper, are crucial to ensure continued provision of high-quality training, particularly in light of a long-term co-existence with the virus.

Limitations of the study were consistent with those often seen in survey-based questionnaires. The response rate has yielded a relatively small sample size but is in keeping with expectations of online surveys. The survey was also not able to discriminate or pair responses that were arising from one center with multiple residents, or residents whose supervisors also responded,

For Peer Review

to allow for direct comparison of perceptions implemented at one institution. This may have also resulted in overlap of responses, causing a falsely high response rate to certain questions. Responses presented in the survey could also be affected by a negative bias with respondents who have concerns or problems with their residency being more likely to complete the survey. Therefore, the concerns raised may be unique to an individual and less applicable to a larger audience. This is particularly important to consider with regards to the free text responses. Differences in the management of the COVID-19 pandemic by individual governments mean that regional and international variations likely exist and must be considered by the reader when applying the findings to their particularly setting. Due to the anonymous nature of the survey and availability through social media platforms, it is conceivable that unscrupulous individuals may have completed the survey more than once or may have not been a resident or supervisor. Finally, this study did not feature a negative control to assess the perception of residency training prior to COVID-19. It is plausible that insufficiencies in training may have been present, but only now have been brought to light. Additional studies are imminently needed to continue assessing the ongoing impact of this global crisis on the veterinary profession in general and training in particular. Longitudinal studies assessing the long-term effect of educational disruption would also ensure that the training of tomorrow's specialists is not hampered and the excellent provision of care to animals can be continued in the wake of COVID-19.

Whilst this study has focused on the specialist training of ECVS small animal surgical residents, we hope that the findings of this study can be made applicable to readers in different educational settings, from those in other specialist disciplines, those undertaking certificate level studies and even undergraduate veterinary and veterinary nursing training.

For Peer Review

In conclusion, the COVID-19 pandemic has provided a unique challenge to small animal surgical residency training. Open communication between supervisors and residents remains key to ensuring a safe and effective working environment. Additionally, the implementation of ancillary training tools through modern digital platforms may help to preserve skills and allow clinical experience to be gained during times of decreased clinical activity. Some of the suggestions presented here, including the use of videography and case-based presentations, may provide a great medium for learning even after the pandemic.

For Peer Review

Acknowledgements

The authors would like to thank REDACTED. for his assistance with statistical review and presentation of the results. Authors 1 and 2 were involved in the conception and design of the study, including acquisition, analysis, and interpretation of the data. They drafted and revised the manuscript and gave approval of the version to be published.

Declaration

The authors declare no conflict of interest related to this report.

For Peer Review

References

1. Caruana EJ, Patel A, Kendall S, Rathinam S. Impact of coronavirus 2019 (COVID-19) on training and well-being in subspecialty surgery: A national survey of cardiothoracic trainees in the United Kingdom. *J Thorac Cardiovasc Surg*. 2020;(January).

2. Shah JP. The impact of COVID-19 on Head and Neck surgery, education, and training. *Head Neck*. 2020;42(6):1344-1347. doi:10.1002/hed.26188

3. Dedeilia A, Sotiropoulos MG, Hanrahan JG, Janga D, Dedeilias P, Sideris M. Medical and surgical education challenges and innovations in the COVID-19 era: A systematic review. *In Vivo (Brooklyn)*. 2020;34:1603-1611. doi:10.21873/invivo.11950

4. Haffer H, Schömig F, Rickert M, et al. Impact of the COVID-19 Pandemic on Orthopaedic and Trauma Surgery in University Hospitals in Germany: Results of a Nationwide Survey. *J Bone Joint Surg Am*. 2020;102(14):e78. doi:10.2106/JBJS.20.00756

5. Zingaretti N, Contessi Negrini F, Tel A, Tresoldi MM, Bresadola V, Parodi PC. The Impact of COVID-19 on Plastic Surgery Residency Training. *Aesthetic Plast Surg*. Published online 2020. doi:10.1007/s00266-020-01789-w

6. Ferrara M, Romano V, Steel DH, et al. Reshaping ophthalmology training after COVID-19 pandemic. *Eye*. Published online 2020. doi:10.1038/s41433-020-1061-3

7. RCVS. Coronavirus: RCVS and BVA issue joint guidance on 'key worker' status for veterinary surgeons. Published 2020. https://www.rcvs.org.uk/news-and-views/news/coronavirus-rcvs-and-bva-issue-joint-guidance-on-key-worker/

8. Gortázar C, de la Fuente J. COVID-19 is likely to impact animal health. *Prev Vet Med*. 2020;180(April):105030. doi:10.1016/j.prevetmed.2020.105030

For Peer Review

9. Singleton DA, Noble PJ, Brant B, Pinchbeck GL, Radford AD. Social distancing impact on companion animal practice. *Vet Rec*. 2020;186(18):607-608. doi:10.1136/vr.m2271

10. Becerra-Flores M, Cardozo T. SARS-CoV-2 viral spike G614 mutation exhibits higher case fatality rate. *Int J Clin Pract*. 2020;74(8):0-2. doi:10.1111/ijcp.13525

11. Grubaugh N, Hanage W, Rasmussen A. Making Sense of Mutation: What D614G Means for the COVID-19 Pandemic Remains Unclear. *Cell Press*. 2020;182(August):794-795.

12. Wise J. Covid-19: New coronavirus variant is identified in UK. *BMJ*. 2020;371:m4857. doi:10.1136/bmj.m4857

13. Ali I. COVID-19: Are we ready for the second wave? *Disaster Med Public Health Prep*. 2020;2019:2019-2021. doi:10.1017/dmp.2020.149

14. Giorgi G, Lecca LI, Alessio F, et al. COVID-19-Related Mental Health E ff ects in the Workplace : A Narrative Review. (December 2019):1-22.

15. Dye TD, Alcantara L, Siddiqi S, et al. Risk of COVID-19-related bullying, harassment and stigma among healthcare workers: An analytical cross-sectional global study. *BMJ Open*. 2020;10(12):1-15. doi:10.1136/bmjopen-2020-046620

16. Azlan AA, Hamzah MR, Sern TJ, Ayub SH, Mohamad E. Public knowledge, attitudes and practices towards COVID-19: A cross-sectional study in Malaysia. *PLoS One*. 2020;15(5):1-15. doi:10.1371/journal.pone.0233668

17. Rashedi J, Mahdavi Poor B, Asgharzadeh V, et al. Risk Factors for COVID-19. *Le Infez Med*. 2020;28(4):469-474.

18. Kabachinski J. Unified communications in healthcare. *Biomed Instrum Technol*. 2011;45(3):234-237. doi:10.2345/0899-8205-45.3.234

For Peer Review

19. Loeb J. What are the issues stalling telemedicine? *Vet Rec*. 2019;185(10):286-287. doi:10.1136/vr.l5534

20. Limb M. Telemedicine: Are we nearly there yet? *Vet Rec*. 2018;182(20):564-565. doi:10.1136/vr.k2187

21. RCVS. RCVS review of the use of telemedicine within veterinary practice Summary Analysis. 2018;(March). https://www.rcvs.org.uk/document-library/telemedicine-consultation-summary/

22. Jumreornvong O, Yang E, Race J, Appel J. Telemedicine and Medical Education in the Age of COVID-19. *Acad Med*. 2020;XX(X):1838-1843. doi:10.1097/ACM.0000000000003711

23. Harendza S, Gärtner J, Zelesniack E, Prediger S. Evaluation of a telemedicine-based training for final-year medical students including simulated patient consultations, documentation, and case presentation. *GMS J Med Educ*. 2020;37(7):1-8. doi:10.3205/zma001387

24. Lee MS. Integrating Telemedicine Into Training : Adding Value to Graduate Medical Education Through Electronic Consultations. *J Grad Med Educ*. 2019;(June):251-254.

25. CM Research. CM Research Corona Virus Tracker Wave 4: What is the continued impact of COVID-19 on the veterinary industry. 2020;(May).

26. Diesen DL, Erhunmwunsee L, Bennett KM, et al. Effectiveness of laparoscopic computer simulator versus usage of box trainer for endoscopic surgery training of novices. *J Surg Educ*. 2011;68(4):282-289. doi:10.1016/j.jsurg.2011.02.007

27. Kilkenny JJ, White K, Singh A. Evaluating veterinary student skill acquisition on a laparoscopic suturing exercise after simulation training. *Vet Surg*. 2019;48(S1):O66-O73. doi:10.1111/vsu.12930

For Peer Review

28. Alaker M, Wynn GR, Arulampalam T. Virtual reality training in laparoscopic surgery: A systematic review & meta-analysis. *Int J Surg*. 2016;29:85-94. doi:10.1016/j.ijsu.2016.03.034

29. Chick RC, Clifton GT, Peace KM, et al. Using Technology to Maintain the Education of Residents During the COVID-19 Pandemic. *J Surg Educ*. 2020;(January). doi:10.1016/j.jsurg.2020.03.018

30. O'Mahoney PRA, Yeo HL, Lange MM, Milsom JW. Driving Surgical Quality Using Operative Video. *Surg Innov*. 2016;23(4):337-340. doi:10.1177/1553350616643616

31. Thia BC, Wong NJ, Sheth SJ. Video recording in ophthalmic surgery. *Surv Ophthalmol*. 2019;64(4):570-578. doi:10.1016/j.survophthal.2019.01.005

32. Saun TJ, Zuo KJ, Grantcharov TP. Video Technologies for Recording Open Surgery: A Systematic Review. *Surg Innov*. 2019;26(5):599-612. doi:10.1177/1553350619853099

33. Giusto G, Caramello V, Comino F, Gandini M. The surgeon's view: Comparison of two digital video recording systems in veterinary surgery. *J Vet Med Educ*. 2015;42(2):161-165. doi:10.3138/jvme.0814-088R1

34. Moshtaghi O, Kelley KS, Armstrong WB, Ghavami Y, Gu J, Djalilian HR. Using google glass to solve communication and surgical education challenges in the operating room. *Laryngoscope*. 2015;125(10):2295-2297. doi:10.1002/lary.25249

35. Munusamy T, Karuppiah R, Bahuri NFA, Sockalingam S, Cham CY, Waran V. Telemedicine via Smart Glasses in Critical Care of the Neurosurgical Patient—COVID-19 Pandemic Preparedness and Response in Neurosurgery. *World Neurosurg*. 2021;145:e53-e60. doi:10.1016/j.wneu.2020.09.076

For Peer Review

472 **Figures and tables**

Figure 1: Perceived Change in Composition of Case Load Seen by Surgical Residents Between March 2020 and August 2020, Compared to Prior to March 2020

473

For Peer Review

Figure 2: Percentage of Residents (n=52) and Supervisors (n=29) That Think Their Institution Has Provided Appropriate Guidance on Working During the Pandemic.

* Denotes statistical significance (P=0.02)

For Peer Review

Figure 3: Percentage of residents that feel safe and well protected from COVID-19 and supervisors that feel that their residents are safe and well protected.

* Denotes statistical significance (P=0.01)

For Peer Review

Table 1: Percentage of respondents that believe their institution has taken measures to preserve the effectiveness of resident training.

* Denotes statistical significance (P=.006)

	Yes	Partially	No
Residents (N=51, 1 – prefer not to answer)	56.9%*	15.7%	27.5%*
Resident Supervisors (N=29)	82.8%*	13.8%	3.45%*

For Peer Review

Table 2: Percentage responses on the effectiveness of teaching methods on the training of surgical residents (n=81)			
	Yes	No	Not Sure
Can remote supervision be an effective part of residency training?	23.5%	49.4%	27.1%
Do virtual clinics and telemedicine help to gain and/or improve clinical skills of residents?	34.5%	42.0%	23.5%
Are web-based teaching sessions, including case presentations, helpful in clinical training?	90.1%	6.17%	3.70%
	Yes	Partially	No
Could we-based teaching replace traditional didactic teaching?	37.0%	42.0%	21.0%
Could live discussion of edited surgical videos be helping in surgical training?	55.6%	39.5%	4.94%
Would simulator training help residents to gain/improve surgical skills?	46.9%	48.1%	4.94%

For Peer Review

Table 3: Perceived impact of COVID-19 on surgical residency training to date and in the future during a long-term period of co-existence with the virus.

	Perceived impact of COVID on residency training to date (n=81)	Perceived impact of COVID on residency training due to phase of co-existence with virus. (n=80)
No impact	11.1%	22.5%
Mild	45.7%	48.8%
Moderate	30.9%	25.0%
Severe	11.1%	3.75%
Catastrophic	1.23%	0%

For Peer Review

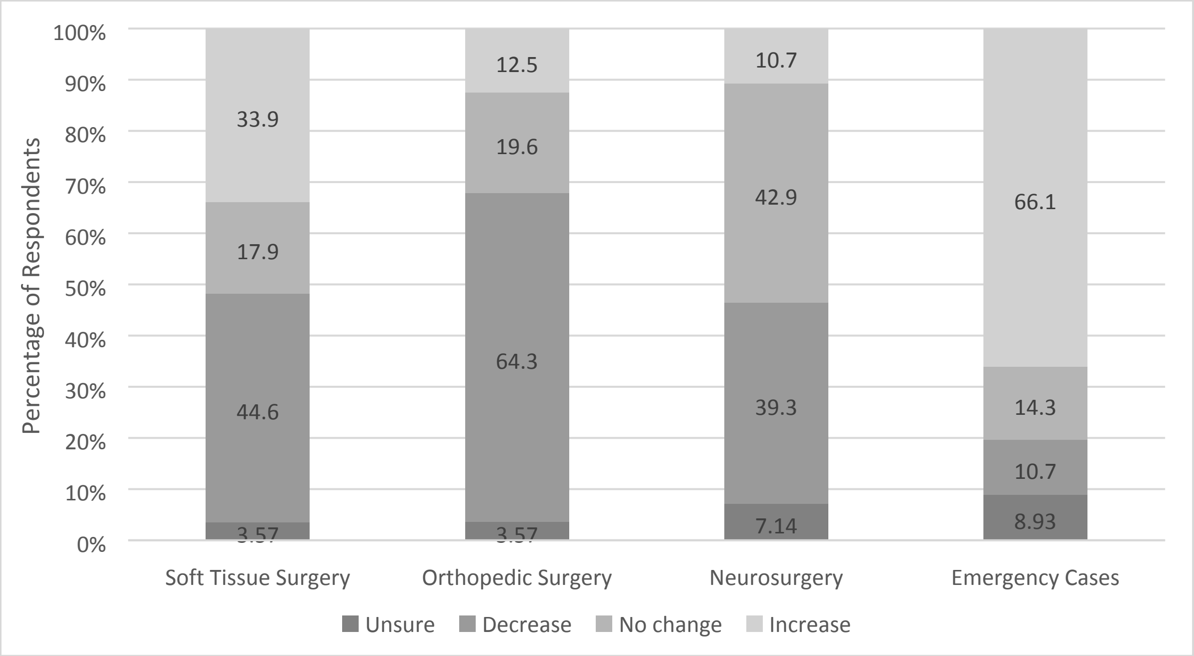

Figure 1: Perceived Change in Composition of Case Load Seen by Surgical Residents and Supervisors Between March 2020 and August 2020, Compared to Prior to March 2020 (n=56).

For Peer Review

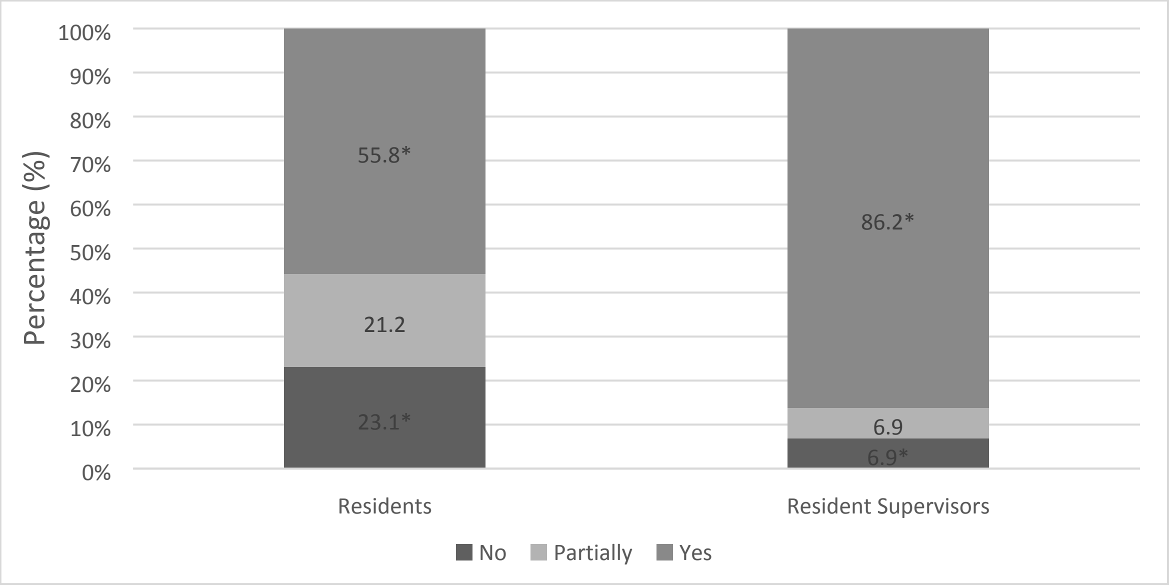

Figure 2: Percentage of Residents (n=52) and Supervisors (n=29) That Think Their Institution has Provided Appropriate Guidance on Working During the Pandemic.

* Denotes statistical significance (P=0.02)

For Peer Review

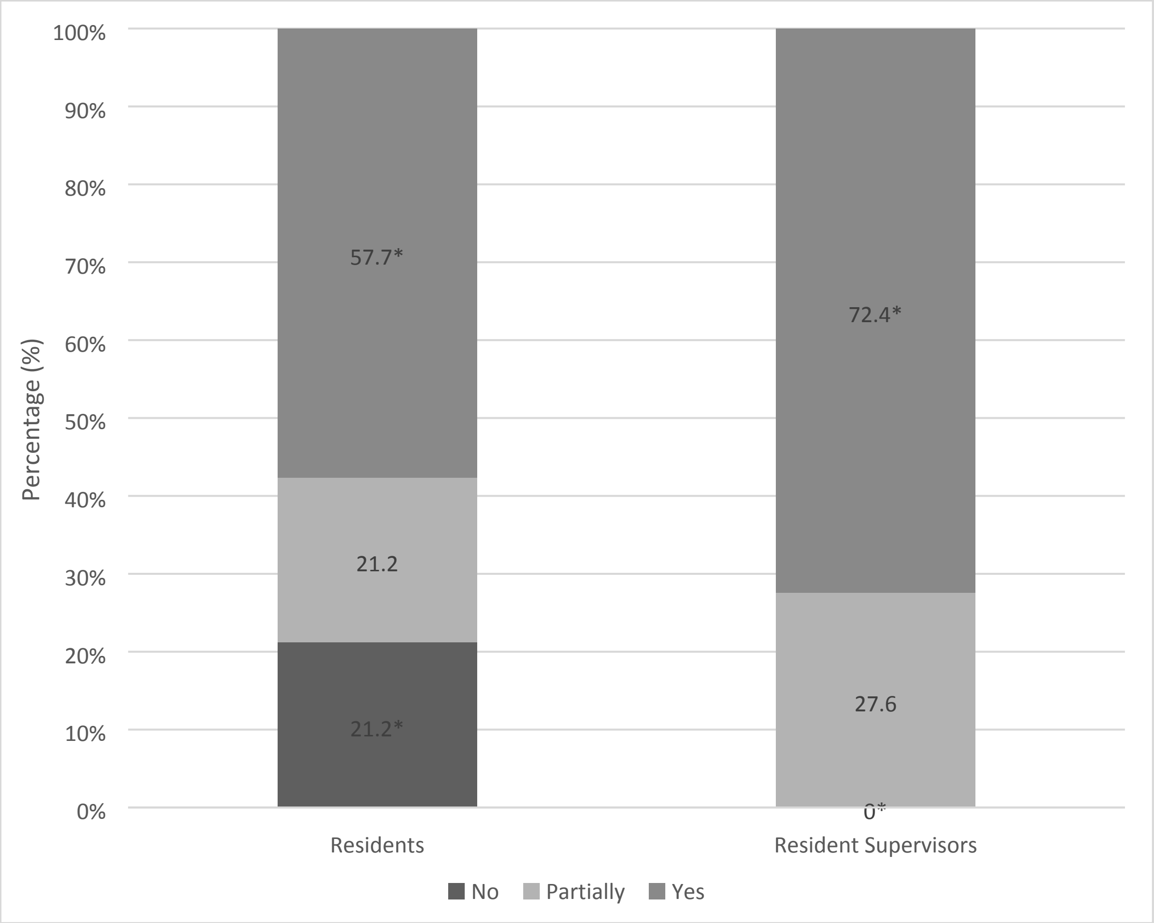

Figure 3: Percentage of residents that feel safe and well protected from COVID-19 and supervisors that feel that their residents are safe and well protected.

* Denotes statistical significance (P=0.01)

Review

Participation Email: Reshaping Surgical Specialist Training in Small Animal Surgery During and After the COVID-19 Pandemic

Dear **XXX,**

We invite all registered residents in small animal surgery and resident supervisors to participate in a short online survey to share your experiences and thoughts on the impact of COVID-19 on surgical training programmes. The survey aims to assess the effect of the pandemic, as well as to explore the methods used to overcome the challenges faced.

This independent research survey is conducted by REDACTED. Results will be made available to the veterinary community via timely scientific publication and are expected to benefit the individual resident and programme supervisor, as well as training institutions and overarching organisations. Please feel free to contact REDACTED with any questions or comments you may have regarding this survey.

Participation will be anonymous and will take no more than 10 minutes to complete. Completion and return of the questionnaire will indicate your willingness to participate in this study. Deadline for responses is Tuesday, 6th October 2020.

Please click this link to start the survey:

[Hyperlink]

Many thanks in advance for your participation, especially during these busy and unprecedented times!

Best wishes,

REDACTED

P.S.: If you would like to receive a summary of our results, please send us an email to REDACTED

Questionnaire A (For Residents):

By completing the survey, I CONFIRM that I am a graduated veterinarian and that I CONSENT to my anonymous responses being processed for the purpose of this project.

Background information and demographics

1) What is your position within the ECVS?
- I am an ECVS registered Resident
- I am an ECVS registered Resident Supervisor
- I am neither ………………(exclusion from study)

2) Please state the type of institution you work at:
- Private
- Academic
- Other, please specify:

3) Please state the type of ECVS residency being undertaken at your institution:
- Standard veterinary surgery residency programme (SVSRP)
- Dual-site/Co-supervised SVSRP
- Alternate veterinary surgery training programme (AVSTP)

4) Please state the total number of ECVS residents at your institution (including yourself)
- Enter value

5) Please enter your year of veterinary degree graduation
- Enter Value

6) Please enter the current year of your residency:
- 1
- 2
- 3
- Other, add free text

7) Please enter your sex:
- Male
- Female
- Prefer not to say
- Other: Free text option

Changes of residency training during COVID-19 pandemic.

8) Has your working rota been changed to COVID-19 pandemic?
- Yes
- No

For Peer Review

9) Have government issued mandates affected the work of veterinarians in your country?
- Yes
- No
- Unsure
- Other: Free text entry

10) Compared to previously, by what percentage do you estimate your surgical activity (cases operated on) changed overall between the months of March 2020 and August 2020?
- No change
- My surgical activity decreased by less than 25%
- My surgical activity decreased by 25–50%
- My surgical activity decreased by 50–75%
- My surgical activity decreased by 75-99%
- My surgical activity decreased by 100%
- My surgical activity did not decrease, but increased by approximately: Free text entry

11) Compared to previously, by what percentage do you estimate your clinical activity (including all cases seen) changed between the months of March 2020 and August 2020?
- No change
- My clinical activity decreased by less than 25%
- My clinical activity decreased by 25-50%
- My clinical activity decreased by 50-75%
- My clinical activity decreased by 75-99%
- My clinical activity decreased by 100%
- My clinical activity did not decrease, but increased by approximately: Free text entry

12) Did the composition of your case load change between March 2020 and August 2020, compared to the type of cases you would have routinely have been expected to see before March 2020?
- Yes
- No
- I am unsure

13) If yes to Q12, How did the composition of your caseload change between March 2020 and August 2020, compared to the type of cases you would routinely have been expected to see before March 2020?

•Soft tissue:
  O More
  o Less
  o Same
  o Comment

•Ortho
  O More

For Peer Review

o Less
o Same
o Comment

•Neuro
O More
o Less
o Same
o Comment

•Emergency
O More
o Less
o Same
o Comment

14) Are you performing any virtual clinic/telemedicine?
- Yes
- No
- Comments

15) Do you feel confident with telemedicine consultations?
- Yes
- No
- Comments

16) In your hospital is there any simulation-based tool available for surgical training?
- Yes
- No
- Comments

17) Have you experienced any gap in the didactic (lecture-based) teaching provided to residents?
- Yes
- No
- Comments

18) Are you attending any internal web-based teaching regularly (journal club, book club etc)?
- Yes
- No
- Still performed in person
- No longer taking place
- Comments

19) Has there been a change in attendance to these sessions if a change to web-based platforms has been made?
- No change
- Increased attendance

For Peer Review

- Decreased attendance
- Unsure
- Comments

20) Have you accessed externally provided web-based teaching (CPD, etc)?
- Yes
- No
- Comments

21) Has there been a change in your attendance to these sessions compared to pre-COVID times?
- No change
- Increased attendance
- Decreased attendance
- Unsure
- Comments

Impact of Changes due to COVID-19

22) Do you think that your institution has provided you appropriate guidance on working during the pandemic?
- Yes
- No
- Not sure
- Comments

23) Do you feel safe and well protected against SARS-CoV-2 working in the hospital/clinic?
- Yes
- Partially (please comment)
- No
- Comments

24) Do you think that the measures taken by your institution to preserve the effectiveness of your training have been appropriate?
- Yes
- Partially (please comment)
- No
- Comments

25) Do you believe that remote supervision could be effective part of residency training?
- Yes
- No
- Not sure
- Comments

For Peer Review

26) Do you believe that virtual clinic and telemedicine may help you to gain and improve your clinical skills?
- Yes
- No
- Not sure
- Comments

27) Do you believe that web-based teaching sessions including case presentations could be helpful to your clinical training?
- Yes
- No
- Not sure
- Comments

28) Do you believe that web-based teaching sessions can replace traditional didactic (lecture-based) teaching?
- Yes
- Only partially
- No
- Comments

29) Do you feel that web-based teaching sessions including live discussion of edited surgical videos could be helpful to your surgical training?
- Yes
- Only partially
- No
- Comments

30) Do you believe that a simulator training may help you to retain and/or improve your surgical skills?
- Yes
- Only partially
- No
- Comments

31) How would you describe the impact of COVID-19 pandemic on your training programme to date?
- No impact
- Mild
- Moderate
- Severe
- Catastrophic
- Unsure
- Comments

For Peer Review

32) Do you think that COVID-19 pandemic will result in an irreversible gap in your training?
- Yes
- Partially (please comment)
- No
- Comments

33) Considering the future long-term restrictions in clinical and surgical activities related to the phase of coexistence with the virus, how do you think will be the impact on your training?
- No impact
- Mild
- Moderate
- Severe
- Catastrophic
- Unsure
- Comments

34) What do you think about an extension to the training programme of your residency?
- Pointless
- Reasonable
- Necessary
- Not sure
- Comments

For Peer Review

Questionnaire B (For Resident Supervisors):

By completing the survey, I CONFIRM that I am a graduated veterinarian and that I CONSENT to my anonymous responses being processed for the purpose of this project.

Background information and demographics

1) What is your position within the ECVS?
- I am an ECVS registered Resident
- I am an ECVS registered Resident Supervisor
- I am neither ………………(exclusion from study)

2) Please state the type of institution you work at:
- Private
- Academic
- Other, please specify:

3) Please state the type of ECVS residency being undertaken at your institution:
- Standard veterinary surgery residency programme (SVSRP)
- Dual-site/Co-supervised SVSRP
- Alternate veterinary surgery training programme (AVSTP)

4) Please state the total number of ECVS residents at your institution:
- Enter value

5) Please enter your year of veterinary degree graduation
- Enter Value

6) Please enter the current year of residency of the resident that you supervise. (Free text can be used for multiple residents supervised):
- 1
- 2
- 3
- Other, add free text

7) Please enter your sex:
- Male
- Female
- Prefer not to say
- Other: Free text option

Changes of residency training during COVID-19 pandemic.

8) Has your working rota of your resident(s) been changed to COVID-19 pandemic?
- Yes
- No

For Peer Review

9) Have government issued mandates affected the work of veterinarians in your country?

- Yes
- No
- Unsure
- Other: Free text entry

10) Compared to previously, by what percentage do you estimate the surgical activity of your resident(s) (cases operated on) changed overall between the months of March 2020 and August 2020?

- No change
- The resident's activity decreased by less than 25%
- The resident's activity decreased by 25–50%
- The resident's activity decreased by 50–75%
- The resident's activity decreased by 75-99%
- The resident's activity decreased by 100%
- The resident's activity did not decrease, but increased by approximately: Free text entry

11) Compared to previously, by what percentage do you estimate the clinical activity of your resident(s) (including all cases seen) changed between the months of March 2020 and August 2020?

- No change
- The resident's activity decreased by less than 25%
- The resident's activity decreased by 25-50%
- The resident's activity decreased by 50-75%
- The resident's activity decreased by 75-99%
- The resident's activity decreased by 100%
- The resident's activity did not decrease, but increased by approximately: Free text entry

12) Did the composition of case load seen by your resident(s) change between March 2020 and August 2020, compared to the type of cases you would have routinely have been expected to see before March 2020?

- Yes
- No
- I am unsure

13) If yes to Q12, How did the composition of your resident(s) caseload change between March 2020 and August 2020, compared to the type of cases they would routinely have been expected to see before March 2020? (If 'No' to Q12, please skip this question)

•Soft tissue:

  O More
  o Less
  o Same

For Peer Review

o Comment

•Ortho

O More
o Less
o Same
o Comment

•Neuro

O More
o Less
o Same
o Comment

•Emergency

O More
o Less
o Same
o Comment

14) Are your resident(s) performing any virtual clinic/telemedicine?

- Yes
- No
- Comments

15) Do your resident(s) feel confident with telemedicine consultations?

- Yes
- No
- Comments

16) In your hospital is there any simulation-based tool available for surgical training of your resident(s)?

- Yes
- No
- Comments

17) Have you experienced any gap in the didactic (lecture-based) teaching provided to your resident(s)?

- Yes
- No
- Comments

18) Are your resident(s) attending any internal web-based teaching regularly (journal club, book club etc)?

- Yes
- No
- Still performed in person
- No longer taking place
- Comments

For Peer Review

19) Has there been a change in attendance to these sessions if a change to web-based platforms has been made?
- No change
- Increased attendance
- Decreased attendance
- Unsure
- Comments

20) Have your resident(s) accessed externally provided web-based teaching (CPD, etc)?
- Yes
- No
- Comments

21) Has there been a change in your attendance to these sessions compared to pre-COVID times?
- No change
- Increased attendance
- Decreased attendance
- Unsure
- Comments

Impact of Changes due to COVID-19

22) Do you think that your institution has provided you appropriate guidance on working during the pandemic to your resident(s)?
- Yes
- No
- Not sure
- Comments

23) Do you feel your resident(s) are safe and well protected against SARS-CoV-2 working in the hospital/clinic?
- Yes
- Partially (please comment)
- No
- Comments

24) Do you think that the measures taken by your institution to preserve the effectiveness of the training of the resident(s) you supervise, have been appropriate?
- Yes
- Partially (please comment)
- No
- Comments

25) Do you believe that remote supervision could be effective part of residency training?
- Yes
- No

For Peer Review

- Not sure
- Comments

26) Do you believe that virtual clinic and telemedicine may help your resident(s) to gain and improve your clinical skills?
- Yes
- No
- Not sure
- Comments

27) Do you believe that web-based teaching sessions including case presentations could be helpful to the training of the resident(s) you supervise?
- Yes
- No
- Not sure
- Comments

28) Do you believe that web-based teaching sessions can replace traditional didactic (lecture-based) teaching?
- Yes
- Only partially
- No
- Comments

29) Do you feel that web-based teaching sessions including live discussion of edited surgical videos could be helpful in the surgical training of the resident(s) you supervise?
- Yes
- Only partially
- No
- Comments

30) Do you believe that a simulator training may help to gain and/or improve the surgical skills of the resident(s) you supervise?
- Yes
- Only partially
- No
- Comments

31) How would you describe the impact of COVID-19 pandemic on the training programme, to date, of the resident(s) you supervise?
- No impact
- Mild
- Moderate
- Severe
- Catastrophic
- Unsure

For Peer Review

- Comments

32) Do you think that COVID-19 pandemic will result in an irreversible gap in the training of the resident(s) you supervise?
- Yes
- Partially (please comment)
- No
- Comments

33) Considering the future long-term restrictions in clinical and surgical activities related to the phase of coexistence with the virus, how do you think will be the impact on the future training of the resident(s) you supervise?
- No impact
- Mild
- Moderate
- Severe
- Catastrophic
- Unsure
- Comments

34) What do you think about an extension of the training programme of your resident(s)?
- Pointless
- Reasonable
- Necessary
- Not sure
- Comments

For Peer Review
